# Arsenic in Soils and Forages from Poultry Litter-Amended Pastures

**DOI:** 10.3390/ijerph8051534

**Published:** 2011-05-12

**Authors:** Shadi Ashjaei, William P. Miller, Miguel L. Cabrera, Sayed M. Hassan

**Affiliations:** Department of Crop and Soil Sciences, The University of Georgia, 3111 Miller Plant Sciences Building, Athens, GA 30602, USA; E-Mails: sashjaei@uga.edu (S.A.); mcabrera@uga.edu (M.L.C.); shassan@uga.edu (S.M.H.)

**Keywords:** poultry litter, fractionation, sodium bicarbonate, residual, water-soluble

## Abstract

In regions of concentrated poultry production, poultry litter (PL) that contains significant quantities of trace elements is commonly surface-applied to pastures at high levels over multiple years. This study examined the effect of long-term applications of PL on soil concentrations of arsenic (As), copper (Cu), Zinc (Zn), and the uptake of these elements by bermuda grass grown on Cecil (well-drained) and Sedgefield (somewhat poorly-drained) soils. The results showed that concentrations of As, Cu, and Zn in soils that had received surface-applied PL over a 14-year period were significantly greater than untreated soil at 0–2.5 and 2.5–7.5 cm depths. However, the levels were well below the USEPA loading limits established for municipal biosolids. Arsenic fractionation showed that concentrations of all As fractions were significantly greater in PL-amended soils compared to untreated soils at 0–2.5 and 2.5–7.5 cm depths. The residual fraction was the predominant form of As in all soils. The water-soluble and NaHCO_3_-associated As were only 2% of the total As. Significant differences were found in concentrations of these trace elements and phosphorus (P) in forage from PL-amended soils compared to that in untreated plots. The concentrations of Cu, Zn, As, and P were significantly greater in forage from Sedgefield amended soil compared to Cecil soil, but were in all cases below levels of environmental concern.

## 1. Introduction

The poultry industry is an important component of agricultural production in Georgia and the other parts of the United States. Poultry litter (PL) is commonly surface-applied to pastures and is considered a valuable source of N, P, and K. It also contains high concentrations of trace elements such as arsenic (As), copper (Cu), and zinc (Zn) as a result of their use as growth promoters in poultry feed. Arsenic occurs in PL as a result of the use of organoarsenical compounds such as roxarsone (ROX, 3-nitro-4-hydroxyphenyl-arsenic acid) as a feed additive at concentrations of 25 to 50 mg kg^−1^ for prevention of fungal disease and for weight gain improvement [1]. Georgia is one of the top broiler chicken producing regions in the United States and due to transport costs, most PL is applied to fields within short distances of poultry houses. Every broiler produces between 1.46 and 2.67 kg of waste over its life span [2,3]. Repeated surface applications of PL could result in accumulation of heavy elements at shallow soil depth due to the immobility of these elements and result in environmental problems. Guptal and Charles [4] reported greater concentrations of As, Cd, Cu, and Mn in fields that had received long-term PL applications compared to unamended soil. Several studies have shown that long-term PL application has an effect on elevation of trace elements (e.g., As, Cu, and Zn) especially at the soil surface [4–8]. A study of the long-term effects of broiler litter applications on Cu and Zn in Maryland Coastal Plain soil showed higher concentrations of these trace elements in PL-amended soil compared to a wooded area [9].

Land application of PL is regulated based on applying N and/or P for crop needs. There are no regulatory standards for trace elements concentrations or loading. In the United States, only municipal biosolids have regulatory limits on trace metals. The USEPA 503 regulation loading limits for As, Cu, and Zn in soil amended with biosolids are 20, 750, and 1,400 mg kg^−1^, respectively [10,11]. These standards are often used as a reference for other land-applied wastes.

Arsenic in soils is distributed among various forms which are associated with different soil constituents. Arsenic fractionation in different soil solid phases can be examined by selective sequential extraction with reagents of increasing dissolution strength. Based on the chemical similarity of P and As, modified versions of P fractionation methods have been adapted for As [12–16] These schemes differ in their sequential extraction steps, reagents, and extraction conditions. A common feature among most methods is extracting with NH_4_Cl or H_2_O (water-soluble As), NaHCO_3_ (surface-adsorbed As), NaOH (bound to Fe oxide), NH_4_F (bound to Al oxide) and the residual phase (acid digest). The results of As fractionation aid in understanding the distribution of As among different soil constituents and help in predicting its mobility and bioavailability. In general, the water-soluble form is considered as readily bioavailable and is presumably susceptible to runoff losses compared to more tightly bound forms. A study of As distribution in PL and long-term PL amended soil (upper coastal plain, MS) that had received PL at a rate of 10 Mg ha^−1^ for 25 years showed that 47% of As in PL was water-soluble fraction. However, 72% of As in PL-amended soils was in the residual fraction and was considered as the least susceptible to runoff losses or downward movement. It was concluded that immediately after PL application there would be a high risk of mobilization of As from top soil, but over time the water-soluble form of As converts to more stable fractions with less susceptibility to be lost by runoff [17].

The objectives of this study were: (1) to estimate the potential environmental concern associated with As, Cu, and Zn in long-term PL-amended pastures; (2) to assess the distribution of different forms of As fractions in PL-amended pastures and (3), to determine forage uptake of As, Cu, Zn, and P in PL-amended pastures. Two southern piedmont soils (well-drained Cecil series and somewhat poorly-drained Sedgefield) were compared in the experiments.

## 2. Materials and Methods

### 2.1. Soil Sampling and Characterization

This research was conducted on six 0.8 ha PL-amended and two control plots with tall fescue (*Festuca arundinacea* Scherb) and bermuda grass (*Cynodon dactylon* L.), located at the College of Agricultural and Environmental Sciences Central Georgia Research and Education Center near Eatonton, GA (39°24′ N, 83°29′ W, elevation 150 m). The soil series comprising the plots include Cecil (fine, kaolinitic, thermic Typic Kanhapludults) and Sedgefield (fine, mixed, active, thermic Aquic Hapludults). Poultry litter had been applied annually to each amended plot since 1995 at 5 Mg ha^−1^ and was applied on April 2008, November 2008, and March 2009 during this experiment. Four PL-amended plots contained predominately Cecil soil, two PL-amended plots contained predominately Sedgefield soil and two unamended (control) plots were largely Cecil soil. Composite soil sampling was conducted in June 2008 two months after PL application with six replicate samples taken from PL-amended plots containing Cecil and Sedgefield soils and 3 replicate samples from control plots (each replicate was a composite of 12 soil subsamples) at 0–2.5 and 2.5–7.5 cm depths. The samples were air-dried and passed through 2-mm sieve, mixed, and stored at room temperature before analysis. Soil particle-size distribution was determined by the micropipette method [18]. Sodium dithionite was used to dissolve total free iron oxide and Tamm’s reagent was used to remove amorphous iron oxide in both PL-amended soils, and Fe was determined by atomic absorption spectrometer [19]. Total carbon was determined on composite samples by dry combustion (Leco CNS analyzer). A 1:1 soil-to-water mixture was used to determine soil pH.

### 2.2. Forage Sampling

Forage from these experimental plots was sampled twice in October 2008, five months after PL application, and in June 2009, three months after PL application. The pastures were predominantly bermuda grass (*Cynodon dactylon L.*) and tall fescue (*Festuca arundinacea Scherb*). Forage samples were taken from two PL-amended plots containing Cecil soil, two PL-amended plots containing Sedgefield soil, and two control plots (containing Cecil soil) with three replicate samples per each plot. Each replicate was a composite sample of 10–12 randomly selected areas of bermuda grass which were clipped manually. The grass was in its vegetative stage approximately 20–30 cm tall, of which the top 10–15 cm was sampled. The samples were oven-dried at 65 °C for 48 hr and ground prior to analysis.

### 2.3. Arsenic Fractionation

Soils were extracted sequentially to determine distinct fractions of soil As using a method described by Shiowatana *et al.* [16]. Soil As pools extracted consist of: (1) water-soluble As (0.01 *M* Ca (NO_3_)_2_); (2) surface-adsorbed As (0.5 *M* NaHCO_3_); (3) Fe- and Al-associated As (0.1 *M* NaOH); and (4) residual As [16]. In the As fractionation, 1 g of air-dried soil was extracted with 30 mL of 0.01 M Ca (NO_3_)_2_ in 30 mL polypropylene high speed centrifuge tubes, shaken for 16 h and centrifuged at 3,000 rpm for 15 min. The supernatant solution was filtered with Whatman No. 42 filter paper. The soil residue was resuspended with 30 mL NaHCO_3_ and shaken for 16 hr, centrifuged as in step 1 and filtered with Whatman No.42 filter paper. The solution was diluted 4-fold prior to analysis in order to run the samples by ICP-MS (Inductively Coupled Plasma Mass Spectrometry, Perkin Elmer Model Elan 9000). In the last step, the soil residue from step 2 was resuspended with 30 mL (0.1 *M*) NaOH for 16 hr, centrifuged as in previous steps and filtered with Whatman No.42 filter paper and diluted 10-fold prior to analysis by ICP-MS. The residual arsenic was calculated by subtracting the sum of all As fractions from total As in soil. The water-soluble As concentrations in PL were determined by extraction of 1 g freeze-dried PL with 10 mL distilled water in 50 mL polypropylene centrifuge tubes. After shaking for 2 h, the extracts were centrifuged at 15,000 rpm for 15 minutes, filtered (0.45 μm), and diluted 20-fold with distilled water prior to analysis by ICP-MS [20].

### 2.4. Total Trace Elements Analysis

Total trace elements in PL-amended soil were determined using the USEPA SW-846 hot plate acid digestion method 3050B [21]. An air-dried soil sample (0.5 g) was transferred to a 250 mL glass Erlenmeyer flask and was covered with a small glass funnel to reflux vapors and placed on a hot plate under a fume hood. Ten mL of 70% HNO_3_ was added to the flask and heated to boiling for about 3 hrs. In order to complete the digestion, 0.5 mL hydrogen peroxide (H_2_O_2_) was added slowly to the flasks after cooling. This addition was repeated until a clear solution was obtained. After cooling, flasks were taken to 100 mL final volume gravimetrically using deionized water and left overnight for settling of particulates. The decanted solutions were stored in polyethylene plastic vials and diluted at a 1:10 ratio prior to analysis by ICP-MS for As, Cu, and Zn. Replicates, blanks, calibration verification, and standard reference material (SRM-2709) were analyzed to ascertain data quality.

Poultry litter samples were freeze-dried (LabConco Lyph. Lock 6, Kansas, MO) and total As, Cu, and Zn in PL was analyzed using a microwave acid digest method USEPA 3051A [22]. All microwave vessels were soaked in 5 % HNO_3_ overnight and cleaned by microwaving with 2 mL HNO_3_ and 10 mL distilled water (CEM corporation, Model 81D) for 20 minutes prior to use for sample digestion. The 0.12 g freeze-dried PL samples were transferred to microwave vessels and 5 mL 70% HNO_3_ was added. Vessels were weighted before and after digestion to assure negligible solution loss during digestion. After removing from the microwave, vessels were cooled to room temperature and 25 mL distilled water was added to all vessels. The final samples were stored in polyethylene vials prior to analysis by ICP-MS. Trace elements (As, Cu, and Zn) and phosphorus concentrations in forage were determined by the same method using 0.1 g oven-dried forage samples.

### 2.5. Mehlich-I (Double Acid) Extractable Cu and Zn

Extractable Cu and Zn in soils at two depths (0–2.5, 2.5–7.5 cm) were measured by the Mehlich-I method. The Mehlich-I extraction solution consists of 0.05 *M* HCl and 0.0125 *M* H_2_SO_4_ which was prepared by adding 4.17 mL of concentrated HCl (12 *M*) and 0.70 mL of concentrated H_2_SO_4_ (17.8 *M*) and brought to volume in a 1 litter volumetric flask. Five g of air-dried soil along with 20 mL of Mehlich-I extraction solution was transferred to 50 mL polypropylene centrifuge tubes and shaken for 5 min [23]. The solution was filtered through Whatman No.42 filter paper. The filtered samples were diluted 1:100 prior to analysis by ICP-MS.

### 2.6. Statistical Analysis

The experimental design was a one-way analysis (treated *vs.* control) with unequal replication and repeated measures. Arsenic fractionation in control and PL-amended soils at 0–2.5 and 2.5–7.5 cm depths were examined using the PROC GLM procedure [24]. The main effect of treatment (control (Cecil) and PL-amended Cecil and Sedgefield soils) on concentrations of trace elements in forage tissue was significant at *p* < 0.0001 and means were separated using LSMEANS procedure with PDIFF option. Mean concentration of trace elements in forage tissue were examined using the PROC MIXED procedure and means were separated using LSMEANS procedure with PDIFF option.

## 3. Results and Discussion

### 3.1. Total Trace Elements in Soils

Total concentrations of As, Cu, and Zn in PL applied to the pasture on April 2008, November 2008, and March 2009 varied with the specific application (Table 1). These variations are due to differences in composition of poultry feeds and PL management practices. Trace element concentrations of As, Cu and Zn were low in all PL samples compared to pollutant limit metal concentrations for land application of biosolids (41 mg As kg^−1^, 1,500 mg Cu kg^−1^, and 2,800 mg Zn kg^−1^) [10].

Table 2 shows the physical and chemical properties of both PL-amended soils. The clay content and total amounts of Fe oxide of amended Cecil soil were greater at both depths compared to those of Sedgefield soil. Hence, it may be presumed that higher Fe content of Cecil soil will increase the soil As retention capacity. Sarkar *et al.* [25] reported that soils with higher concentrations of amorphous Fe/Al oxides retain more As and thus reduce its bioavailability. Rutherford *et al.* [26] found a strong correlation between acid-extractable Fe and As content of PL-amended soils, suggesting adsorption of As with Fe oxides.

There were differences in total concentrations of As, Cu, and Zn in PL-amended soils compared to those in control plot at 0–2.5 cm and 2.5–7.5 cm depths (Table 3). Total concentration of As was greater in PL-amended Cecil and Sedgefield soils (3.67 mg kg^−1^ and 3.91 mg kg^−1^) compared to those in control plots (1.46 mg kg^−1^) at 0–2.5 cm depth. Similar results were found for total As concentrations in both PL-amended soils compared to those in control plots at 2.5–7.5 cm depth. Han *et al.* [17] also found significant amounts of As in PL-amended soils with a long-term history of PL application (25 years) compared to unamended soil, and total As concentrations in the amended soils were three times greater than those in unamended soils (8.4 *vs.* 2.68 mg kg^−1^). The concentrations of As in Cecil and Sedgefield soils were not different compared with each other at either depth. Total Cu and Zn were greater in both PL-amended soils compared to those in control plots at the 0–2.5 cm depth, although not at the lower depth (Table 3). Han *et al.* [27] reported concentrations of Cu and Zn in unamended soil (0–10 cm) were significantly lower (2.2 and 9.8 mg kg^−1^) compared to PL-amended soil (74.5 and 88.8 mg kg^−1^). Brock *et al.* [28] also showed accumulation of Cu and Zn in soils with a history of 40 years of PL application in the plow layer (0–17.5 cm). The total Cu concentrations ranged from 5.9 to 30.1 mg Cu kg^−1^ soil, and total concentrations of Zn were up to 112 mg Zn kg^−1^ soil. Other studies reported elevation of Cu and Zn concentration in the top 5–10 cm depth in short and long-term application of PL [27,29]. Litter applications for 15 to 25 yr resulted in accumulation of Cu and Zn to a depth of 40 cm compared with unamended soil [5].

The observed distribution of trace elements is clearly due to the surface application of the PL and lack of incorporation into the A horizon. However, concentrations of trace elements were well below USEPA loading limits at both depths. For an application rate of 5 Mg ha^−1^ per year with an average As concentration of 20 mg kg^−1^ in PL applied on the soil surface, the average As input rate was approximately 0.1 kg As ha^−1^ yr^−1^. This annual As loading rate is below the annual ceiling rate (2.0 kg As ha^−1^ yr^−1^) for safe land application of biosolids [10]. The same estimation can be done for Cu and Zn with average concentrations of 500 mg Cu kg^−1^ and 400 mg Zn kg^−1^ in PL applied at a rate of 5 Mg ha^−1^ per year. The Cu and Zn input rates were approximately 2.5 kg Cu ha^−1^ yr^−1^ and 2.0 kg Zn ha^−1^ yr^−1^ which are below the annual ceiling rates (75 kg Cu ha^−1^ yr^−1^ and 140 kg Zn ha^−1^ yr^−1^) for safe land application of biosolids.

### 3.2. Sequential Extraction of As

Sequential extraction was used here in the operational sense of assessing the solubility of As in response to increasingly harsh chemical extractants that have been correlated with specific types of As binding in soils. In the sequential extraction data, analysis of variance showed that there was a statistical difference in main effect of treatment (control and PL-amended Cecil and Sedgefield soils) for all fractions. However, only water-soluble and Fe/Al-associated As showed a significant interaction effect between treatment and depth at *p* < 0.05. There were significant differences in concentrations of water-soluble As in PL-amended soils compared to control at both depths (Table 4). It was calculated that 38%, 25%, and 26% of total As in PL applied in April 2008, November 2008, and March 2009 was in water-soluble form, respectively. Only 2% of total As was in water-soluble fraction two months after PL application in both soils at 0–2.5 cm depth. It was observed that 10% and 13% of total As was in surface adsorbed As (NaHCO_3_) fraction at 0–2.5 cm depth in PL-amended Cecil and Sedgefield soils. In addition, the amount of NaHCO_3_-associated As was significantly greater in Sedgefield soil compared to Cecil soil only at depth of 0–2.5 cm. Solubility of As in PL-amended soils, indicated here by water-soluble and NaHCO_3_-associated As, is linked to its mobility and plant uptake. The fact that long-term PL-amended Sedgefield soil has more NaHCO_3_-associated As suggests a greater potential loss of As via runoff into surface water systems or uptake by plants compare to that in PL-amended Cecil soil. Iron and Al-associated As (NaOH) in two PL-amended soils were significantly greater than control at both depths. There was no significant difference in concentration of Fe/Al-associated As between Cecil and Sedgefield soils, which may suggest As was associated with amorphous Fe oxides, which were present at similar levels in both soils (Table 2). The residual fraction was the major form of As with approximately 50% of total As in both amended soils. Overall, there were no significant differences in concentrations of As fractions between PL-amended soils at 0–2.5 and 2.5–7.5 cm except for NaHCO_3_-associated As at 0–2.5 cm depth. Apparently, As transforms to more stable forms, resulting in decreased As bioavailability and mobility months after PL application. This may also indicate possible loss of water-soluble As via runoff shortly after PL is applied to field.

### 3.3. Plant Uptake by Forage

The results of statistical analysis by ANOVA of trace elements concentrations in forage tissue from PL-amended soil and control showed that there was a significant main treatment effect of P, Cu, Zn, and As concentrations in forage tissues from control and PL-amended soils (Table 5). However, no significant interaction was found between treatment and date effect for any trace elements or P (October and June sampling). There were differences in concentrations of all trace elements in forage tissue from PL-amended soils compared to those in control plot except for amount of Zn in forage tissue from Cecil soil (Table 6). Another study of metal uptake by tall fescue as affected by PL application at low (5.6 t ha^−1^) and high (11.3 t ha^−1^) rates showed that Zn and Cu uptake were significantly greater in PL-amended soil compared to the control [30]. However, Kingery *et al.* [5] showed that tall fescue grown in pastures with a history of long-term litter application had similar Cu and Zn concentrations as fescue grown in pastures with no history of litter application. The concentration of As in forage from Sedgefield soil was significantly greater than Cecil soil. The coarse-textured soils likely contain higher amounts of readily mobile As, while As in fine textured soils is mainly immobile due to higher content of minerals and organic constituents which are capable of binding anionic As species. There were no differences in concentrations of Cu and Zn in forage tissues from Cecil soil compared to those from Sedgefield soil.

The concentrations of Cu and Zn in the forage samples did not exceed critical phytotoxic levels reported by Macnicol and Beckett [31] (21–40, and 210–560 mg kg^−1^ for Cu, and Zn, respectively). Long-term PL application did not produce tissue Cu and Zn levels above the NRC (National Research Council) maximum tolerance level of Cu for cattle (40 mg Cu kg^−1^ feed) or for sheep (15 mg Cu kg^−1^ feed); similarly, Zn was well below maximum levels for cattle (500 mg Zn kg^−1^ feed) and sheep (300 mg Zn kg^−1^ feed) [32]. The forage samples from all soils were in fact Cu deficient for animal feed based on the minimum recommended Cu concentration for cattle rations of 9 mg Cu kg^−1^ [33]. Soon *et al.* [34] reported small increases in the Cu content of corn grain and bermudagrass grown in soil amended with sewage sludge applications (31 kg Cu ha^−1^) for five consecutive years.

The minimum recommended Zn content in cattle rations average 35 mg kg^−1^; forage samples from both PL-amended soils were close to that amount and considered Zn-sufficient for animal feed. Warman and Termeer [35] found that Zn content of grass forage and corn tissues grown in soil amended with sewage wastes for two consecutive years (applied twice a year) were below the 35 mg kg^−1^ minimum Zn requirement for cattle. The maximum tolerance levels of As for cattle and sheep are 30 mg As kg^−1^ feed [32]. The data shown in Table 6 indicate that the As content of bermudagrass tissues grown in both PL-amended soils are below that amount. Since only a small fraction of total As was in the water-soluble fraction in PL-amended soils, PL had minor effect on elevation of total As in forage.

The result of Mehlich-I extraction, which is used to assess potentially plant available Zn in soil testing, showed greater concentrations of Cu and Zn in PL-amended Cecil and Sedgefield soils compared to those in control plot at both depths (Table 7). Concentrations of extractable Zn were greater in PL-amended Sedgefield and Cecil soils (47.4 and 47.5 mg kg^−1^) at 0–2.5 cm compared to those in 2.5–7.5 cm depth (10.45 and 11.29 mg kg^−1^). Only Cu concentrations were greater in PL-amended Sedgefield soil at 0–2.5 cm (6.60 mg kg^−1^) compared to that in 2.5–7.5 cm depth (4.60 mg kg^−1^) at *p* < 0.01. The fact that concentrations of extractable Cu and Zn were greater in PL-amended soils compared to those in control plot appear to be linked with the greater amounts of Cu and Zn in bermudagrass tissue from PL-amended soils and compared to that from control plots. However, soil test Zn and Cu increased much more than tissue levels with PL amendment. Zhou *et al.* [36] reported significant linear correlation between extractable soil Zn (and Cu) concentrations extracted by 1.0 *M* NH_4_NO_3_ and Zn (and Cu) concentrations in radish tissues. Warman and Termeer [35] found that only Mehlich-I Cu was highly correlated with forage tissue Cu (*r* = 0.98), and the correlation coefficients for extractable Zn and Zn uptake were not significant in soil with biosolids applications.

## 4. Conclusions

Total concentrations of As, Cu, and Zn in soils that had received long-term surface-applied PL were significantly greater than in unamended soil. If the USEPA annual loading limits are used as a frame of reference for serious environmental hazard, the concentrations were well below those limits. Arsenic fractionation showed concentrations of all As fractions were significantly greater in PL-amended soils compared to controls at 0–2.5 and 2.5–7.5 cm depths. However, there were no significant differences in concentrations of any As fractions between two PL-amended soils at either depth except for NaHCO_3_-associated As at 0–2.5 cm depth. The greater amounts of NaHCO_3_-associated As seen in the somewhat poorly-drained Sedgefield soils amended with PL suggests greater potential loss of As via runoff or uptake by plants for these soils compared to PL-amended Cecil soils. Arsenic in the PL-amended soils was dominantly found in the residual fraction which is the least susceptible fraction to runoff losses as soluble As or downward movement.

There were greater concentrations of As, Cu, Zn, and P in forage from PL-amended soils compared to those from control. Although PL application resulted in elevated concentrations of As, Cu, Z, and P in forage tissues, it did not cause tissue As, Cu, and Zn levels to increase above the NRC domestic animal mineral tolerance levels. The forage samples from both PL-amended soils were Zn sufficient and Cu deficient for animal feed.

## Figures and Tables

**Table 1.** Total concentrations (mg kg^−1^) of trace elements in poultry litter applied on April 2008, November 2009, and March 2009.

	**As**	**Cu**	**Zn**
**April 2008**	23.6	207	229
**November 2008**	14.9	743	431
**March 2009**	26.7	672	671

**Table 2.** General chemical and physical properties of Cecil and Sedgefield PL-amended soils at 0–2.5 and 2.5–7.5 cm depths.

**Soil**	**Depth (cm)**	**pH (1:1 water)**	**Composition (mg kg ^−1^)**			**Particle size distribution (%)**		

			**Total Fe Oxide**	**Amorphous Fe Oxide**	**Total C**	**Sand**	**Silt**	**Clay**
Cecil	0–2.5	6.00	7,173	3,397	57.4	58	34	8
Cecil	2.5–7.5	5.87	7,511	3,356	26.4	49	36	15
Sedgefield	0–2.5	5.99	4,505	3,259	44.7	75	23	2
Sedgefield	2.5–7.5	5.85	5,281	3,936	20.8	72	25	3

**Table 3.** Total concentrations (mg kg^−1^) of As, Cu, and Zn in poultry litter-amended pastures and control plot.

	**As**	**Cu**	**Zn**
Depth (0–2.5 cm)			

Control	1.46 a ^a^	13.6 a	32.0 a
Cecil (PL-amended)	3.67 b	95.6 b	121 b
Sedgefield (PL-amended)	3.91 b	88.2 b	88.0 c

Depth (2.5–7.5 cm)			

Control	1.57 a ^a^	29.3 a	34.0 a
Cecil (PL-amended)	3.04 b	44.1 ab	51.6 a
Sedgefield (PL-amended)	3.46 b	66.5 c	56.0 a

USEPA loading Limit	20	750	1,400

a Means within a column at a given depth followed by the same letter are not different significantly according to LSD (*p* < 0.05)

**Table 4.** Arsenic fractionation (mg kg^−1^) in poultry litter-amended soils at 0–2.5 and 2.5–7.5 cm depths.

	**WE^a^**	**NaHCO_3_**	**NaOH**	**Residual**	**Total**
Depth (0–2.5 cm)					

Control	0.03 a ^b^	0.17 a	0.55 a	0.55 a	1.29 a
Cecil (PL-amended)	0.07 b	0.36 b	1.43 b	1.81 b	3.67 b
Sedgefield (PL-amended)	0.07 b	0.53 c	1.52 b	1.79 b	3.91 b

Depth (2.5–7.5 cm)					

Control	0.003 a ^b^	0.07 a	0.81 a	0.50 a	1.39 a
Cecil (PL-amended)	0.017 b	0.21 ab	1.09 b	1.68 b	3.03 b
Sedgefield (PL-amended)	0.014 b	0.24 b	1.19 b	2.00 b	3.46 b

a WE, NaHCO3, and NaOH stand for water-soluble-, adsorbed-, and Fe/Al-associated arsenic, respectively;

b Means within column followed by the same letter are not different significantly according to LSD (*p* < 0.05).

**Table 5.** Analysis of variance of P, Cu, Zn, and As concentrations (P > F) in bermuda grass samples from poultry litter-amended soils.

**Source**	**P**	**Cu**	**Zn**	**As**
Treatment ^a^	0.028	0.0012	0.034	0.003
Date	0.26	0.0003	0.045	0.75
Treatment*Date ^b^	0.95	0.25	0.76	0.79

a Main treatment effect (forage samples grown on control (Cecil) and PL-amended Cecil and Sedgefield soils) significant at *p* < 0.05;

b Treatment by date interaction (significant at *p* < 0.05).

**Table 6.** Concentrations of elements (mg kg^−1^) in bermuda grass tissue from control and poultry litter-amended soils.

	
	**P**	**Cu**	**Zn**	**As**
Control	2,189 a ^a^	5.10 a	25.7 a	0.16 a
Cecil (PL-amended)	2,746 b	7.05 b	34.2 ab	0.23 b
Sedgefield (PL-amended)	2,927 b	7.23 b	41.3 b	0.31 c

a Means within a column followed by the same letter are not different significantly according to LSD (*p* < 0.05).

**Table 7.** Mehlich-I extractable Cu and Zn (mg kg^−1^) in control and poultry litter-amended soils.

	**Cu**	**Zn**
Depth (0–2.5 cm)		

Control	0.54 a ^a^	6.56 a
Cecil (PL-amended)	3.71 b	47.4 b
Sedgefield (PL-amended)	6.60 c	47.5 b

Depth (2.5–7.5 cm)		

Control	0.98 a ^a^	3.14 a
Cecil (PL-amended)	3.70 b	11.29 b
Sedgefield (PL-amended)	4.60 b	10.45 b

a Means within a column in a given depth followed by the same letter are not different significantly according to LSD (*p* < 0.05).
